# Effects of smokeless nicotine on blood physiology, biochemical, and histological alterations using* Labeo rohita* as a model organism

**DOI:** 10.5455/javar.2024.k796

**Published:** 2024-06-19

**Authors:** Shabbir Ahmad, Hasnain Akmal, Sajid Ali, Kamran Jafar, Muhammad Shoaib, Muqadas Shahzadi, Iqra Akram, Taqi Shahid Jaffari, Irfan Ahmad, Arva Mehmood, Khurram Shahzad

**Affiliations:** Department of Zoology, Faculty of Life Sciences, University of Okara, Okara, Pakistan

**Keywords:** Nicotine, biochemistry, lipid profile, hematology, histology, *Labeo rohita*

## Abstract

**Objective:**

The present research was conducted to evaluate the negative effects of nicotine powder on the blood physiology, and biochemical and histological alterations of *Labeo rohita.*

**Materials and Methods:**

Fish were divided into four groups (1–4). Fish groups 2, 3, and 4 were exposed to different concentrations of nicotine, such as 0.75, 1.25, and 1.75 mg/l, while group 1 acted as a control. To find out the long-term impact of nicotine on body physiology, we conducted a 42-day experiment. After the completion of the experiment, hematology, biochemical assays, and histology were done.

**Results:**

Results revealed a considerable increase in HGB, red blood cells, WBCs, hematocrit, mean corpuscular volume, red cell distribution width -SD, procalcitonin, neutrophils, lymphocytes, monocytes, triglycerides, total cholesterol, low-density lipoprotein, very low-density lipoprotein, alanine transaminase, aspartate aminotransferase, globulin, thyroid stimulating hormone, BUN, creatinine, and blood glucose levels, whereas mean corpuscular hemoglobin concentration, mean corpuscular hemoglobin, RDW, platelet, high-density lipoprotein, albumin, total proteins, and T_3_ levels were significantly (*p* ≤ 0.05) decreased in exposed fish as compared to control group fish. Histological alterations showed that exposure to smokeless nicotine causes deleterious and degenerative effects in the liver, kidney, and gills of exposed fish.

**Conclusion:**

Nicotine administration in fish results in adverse effects on different biochemical and hematological parameters and causes histological alterations in some vital organs of exposed fish.

## Introduction

Nicotine and tobacco delivery products have been used in the past decade as alternatives to traditional tobacco products that are less harmful, e.g., tobacco heating products, e-cigarettes, and oral nicotine pouches [1]. Nicotine is the most frequently used abusive drug, having primary psychoactive effects [2]. Oral nicotine pouches and chewing tobacco are also counted as consumption [3]. Oral pouches have nicotine and flavorings added to a non-tobacco substrate and a permeable pouch material. This reduces the risk of further health risks for consumers. Azzopardi et al. [4] cite recent chemical analyses that demonstrate MOPs have substantially fewer pollutants than cigarettes. These are available for purchase, for instance, through LYFT and VELO, though this may vary depending on the Pakistani market. According to Slotkin [5], much of the harm that comes from smoking comes from the neurotoxic nicotine, which is found in MOPs. Numerous studies have been conducted on various cell types, as well as on animals and humans, to further understand the effects of nicotine [6].

Nicotine can cause a variety of unpleasant physical effects, including bloating, hunger, and a slow heart rate [7]. Smokeless nicotine is less harmful than cigarettes, nevertheless, the majority of tobacco consumers use a moist oral smokeless tobacco product, rather than cigarettes [8]. An increase in blood pressure, oxygen demand in the heart, and lipid metabolism are all caused by nicotine’s stimulation of the adrenergic drive [9]. Recent studies have found that nicotine contributes to an atherogenic lipid profile [10]. Nicotine is the main cause of atherosclerosis, coronary heart disease, liver damage, and increased free fatty acids, triglycerides, and very low-density lipoprotein (VLDL). More catecholamines stimulate the sympathetic nervous system and lipolysis [11].

Various animal models have been utilized to investigate behavioral disruptions, such as stress, linked to prolonged drug exposure, and relapse occurrences. Most of these investigations have been conducted on rodents, specifically rats and mice. However, there is also data available from studies conducted on other animal species, such as monkeys, zebrafish, and canines, as well as from human volunteers, as reported by Świątkowski et al. [12]. The primary goals of this study were to investigate the impact of smokeless nicotine on several aspects of the hematological parameters, lipid profile, kidney function, liver enzymes, metabolism, and histological changes in different organs of the freshwater fish *L. rohita*.

## Materials and Methods

### Ethical approval

The current research obtained ethical approval from the University of Okara’s Ethical Committee (Reference number: UO/ETH/2023/misc.).

### Animal collection and placement

Fish were taken from a fish farm at Head Balloki, District Kasur, Punjab, Pakistan, and transported to the university aquaculture laboratory; no mortality was found during transport. Fish with an average size of 15.48 ± 0.202 cm and weighing 26.31 ± 5.63 gm were placed in a glass aquarium with dimensions of 20′′ × 30′′ × 30′′ cm and 40 l of water. The fish were rinsed with a 0.1% KMnO_4_ solution and acclimatized in glass aquariums for 7 days.

### Experimental design

For the experimental trial, four groups were formed: a control group and three treatment groups, each consisting of three replicas. Each group had twelve fish. Group 1 was designated as a control group for freshwater nicotine-free. Groups 2, 3, and 4 of fish were subjected to varying quantities of nicotine, specifically 0.75, 1.25, and 1.75 mg/l, respectively, for 42 days. These nicotine doses were selected based on a prior study conducted by Nambi et al. [13]. Water quality indicators, including temperature, dissolved oxygen, and pH, were also observed.

### Nicotine extraction

Nicotine oral pouches VELO (USA) were purchased from a local supplier. Aqueous extracts of 6 mg, 10 mg, and 14 mg of nicotine from pouches were prepared. Solution prepared by removing 5 pouches of material; each pouch contains 6 mg, 10 mg, and 14 mg of nicotine, respectively. A conical flask containing 20 ml of complete cell culture media was incubated at 37°C with 150 revolutions per minute shaking for 1 h. A centrifuge was used to separate the solids after the mixture had been shaken, and the supernatant was filtered on 5 and 0.2 μm filter paper [14]. Nicotine powder was dissolved in a water tank by making its stock solution in distilled water.

### Hematology

At the end of the experimental trial, fish (*n = *5 for each group) were taken from the control and treated groups. Total body weight and total body length were measured before blood sampling and dissection. Blood was collected using a BD syringe from the abdominal vein and placed in EDTA and gel vials for hematology and serology. Different hematological parameters, such as red blood cells, white blood cells (WBCs), hemoglobin, hematocrit (HCT), mean corpuscular hemoglobin (MCH), mean corpuscular volume (MCV), and mean corpuscular hemoglobin concentration (MCHC), were calculated by an automatic hematology analyzer (Sysmex XE–5,000).

### Biochemical assay

The plasma was obtained from the blood samples using centrifugation at a force of 9,400 times the acceleration due to gravity for 20 min in a cooling centrifuge. Subsequently, the resulting concentrate was stored at a temperature of 4°C. The protein contents of the samples were determined using the Siddiqui et al. [15] technique, with bovine serum albumin serving as the standard. The glucose concentration was measured via the O-toluidine assay [16]. Creatinine, urea, and BUN levels were determined using kits provided by Biomerieux (France). The activity of alanine transaminase (ALT) and aspartate aminotransferase (AST) was assessed using the method of Lala et al. [17]. The levels of triglycerides, cholesterol, high-density lipoprotein (HDL), low-density lipoprotein (LDL), and VLDL were determined using spectrophotometry, following the method published by Rodríguez et al. [18]. Bayer diagnostics standard kits from Egypt were used for this purpose. Serum T3, tetraiodothyronine (T4), and thyroid-stimulating hormone (TSH) levels were measured using an ELISA kit (USA) per the instructions provided by the manufacturer.

### Histology

Following blood collection, the fish were dissected, and the liver, gills, and kidneys were extracted and stored in a solution of 10% formaldehyde for histological analysis. Following fixation, the tissue samples underwent dehydration using a sequence of alcohol concentrations as the dehydrating agent. Subsequently, the samples were rendered transparent and then embedded in paraffin wax. Tissue sections (4 µm) were sliced using a rotary microtome and stained with hematoxylin and eosin. The prepared slides were analyzed using an optical microscope equipped with a camera.

### Statistical analysis

The statistical analysis was conducted using ANOVA using IBM SPSS (Ver. 21) software at *p < *0.05 level of significance.

## Results

### Hematological analysis

Hematological results showed that red blood cells (RBCs), HGB, WBCs, HCT, MCV, red cell distribution width (RDW)-SD, procalcitonin (PCT), neutrophils, lymphocytes, and monocytes were significantly increased as compared to the nicotine-free group, while MCHC, MCH, RDW, and platelets were significantly decreased, as shown in Table 1.

### Lipid profile

**Figure 1.**
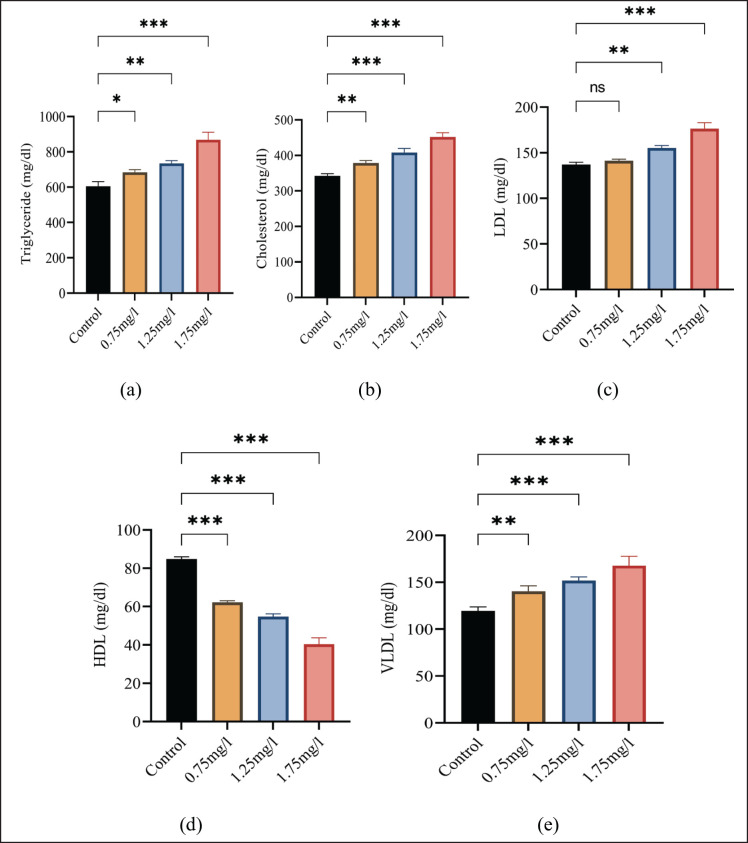
(a) Shows variations in triglycerides among the groups, (b) showing variations in cholesterol among groups, (c) showing variations in LDL among the groups, (d) showing variations in HDL among the groups, and (e) showing variations in VLDL among the groups. All values are mean ± standard deviation (SD) at a 0.05% level of significance.

The statistical values of cholesterol, triglycerides, VLDL, HDL, and LDL are presented in Figure 1. The result of the one-way ANOVA statistic showed a significant (*p* ≤ 0.05) increase in triglycerides, cholesterol, LDL, and VLDL levels in the experimental group as compared to the control group. A significant decrease in HDL was observed.

### Effects on liver enzymes and blood proteins

**Figure 2.**
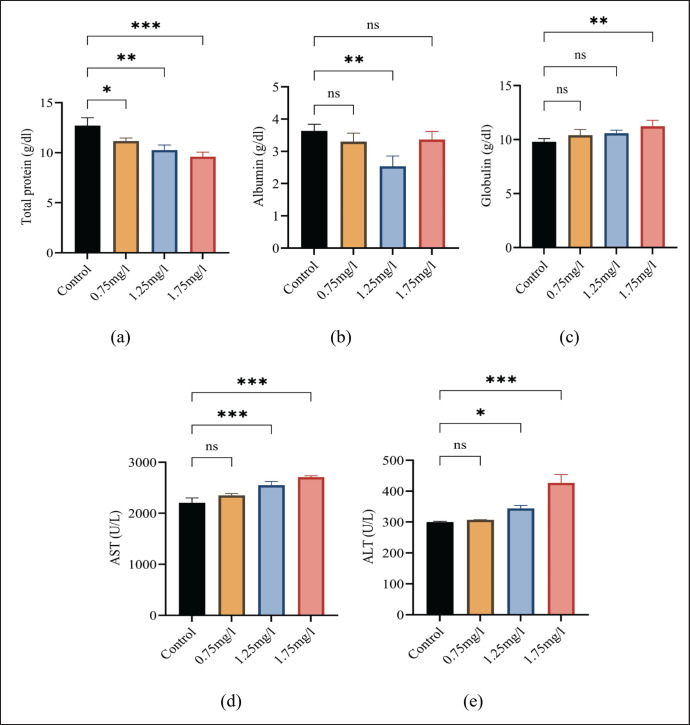
(a) Shows variations in total proteins among the groups, (b) showing variations in albumin among the groups, (c) showing variations in globulin among the groups, (d) showing variations in AST among the groups, and (e) showing variations in ALT among the groups. All values are mean ± standard deviation (SD) at a 0.05% level of significance.

The statistical (mean and SD) values of AST, ALT, albumin, globulin, and total protein are presented in Figure 2. ANOVA statistics revealed a significant (*p *≤ 0.05) increase in ALT, AST, and globulin after exposure to nicotine powder in the treated group as compared to the control group, while a significant (*p* ≤ 0.05) decrease in albumin and total proteins was observed.

### Effects on thyroid hormones

**Figure 3.**
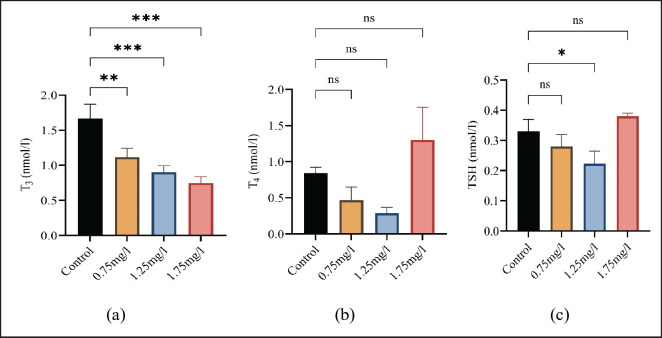
(a) Shows variations in T_3 _among the groups, (b) shows variations in T_4_ among the groups, and (c) shows variations in TSH among the groups. All values are mean ± standard deviation (SD) at a 0.05% level of significance.

The statistical values of triiodothyronine, thyroxine, and thyroid stimulating hormone are shown in Figure 3. The present results showed a significant (*p *≤ 0.05) increase in TSH level in treated groups as compared to the nicotine-free group and a significant (*p* ≤ 0.05) decrease in T_3_ level. However, no significant (ns) decrease or increase was observed in the T4 level.

### Blood glucose and renal effects

**Figure 4.**
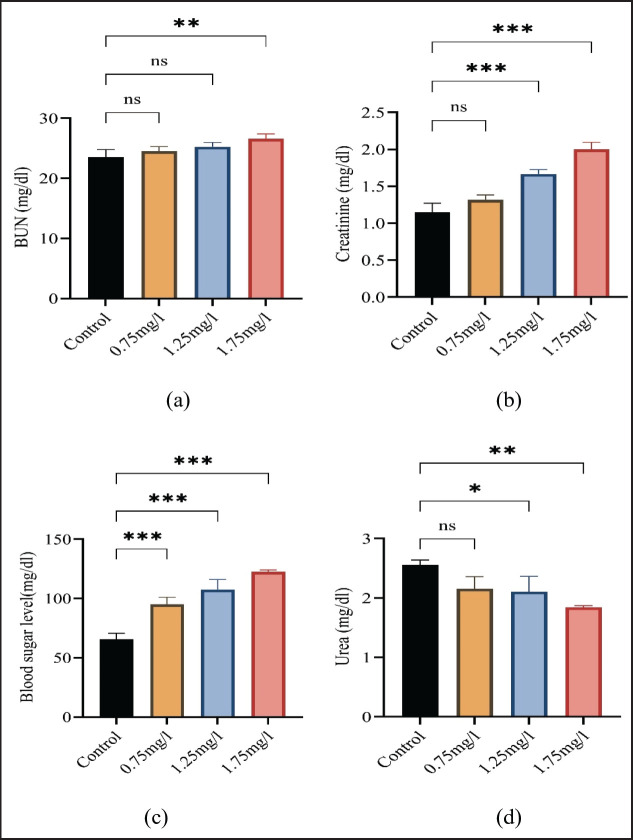
Showing variations in (a) blood urea nitrogen, (b) creatinine, (c) blood sugar, and (d) urea among the groups. All values are mean ± standard deviation (SD) at a 0.05% level of significance.

The mean values of blood sugar, creatinine, urea, and BUN (blood urea nitrogen) are presented in Figure 4. The statistical results showed that the levels of BUN, creatinine, and blood glucose were significantly increased as compared to the nicotine-free group. A significant decrease in urea level was observed.

**Table 1.** Showing the hematological profile of *L. rohita* exposed to different concentrations of nicotine.

Parameters	Control	T1 = 0.75mg/l	T2 = 1.25mg/l	T3 = 1.75mg/l
HGB (g/dl)	5.533 ± 0.404	8.133 ± 0.665*	9.400 ± 0.556*	11.03 ± 0.862*
WBC (×10^3^/µl)	15.07 ± 0.709	13.10 ± 1.153*	17.13 ± 0.450*	18.60 ± 0.40*
RBC (×10^6^/ µl)	1.637 ± 0.465	2.920 ± 0.175	3.333 ± 0.411*	3.027 ± 1.057*
HCT (%)	17.97 ± 2.91	39.93 ± 5.159*	43.60 ± 2 .931*	50.77 ± 1.301*
MCV (Fl)	137.3 ± 3.92	143.5 ± 1.179	133.2 ± 4.922	152.6 ± 2.352*
MCH (pg)	40.03 ± 6.31	33.80 ± 6.026	33.97 ± 4.332	29.23 ± 4.315*
MCHC (g/dl)	101.2 ± 10.71	51.77 ± 28.60*	45.20 ± 19.98*	42.23 ± 20.47*
RDW	23.07 ± 2.60	18.63 ± 3.600	11.07 ± 2.538*	10.17 ± 2.307*
RDW-SD (%)	33.80 ± 4.13	43.07 ± 2.889*	49.57 ± 4.148*	73.93 ± 3.995*
PLT (×10^3^/µl)	203.3 ± 9.85	160.2 ± 10.25*	151.6 ± 6.235*	137.3 ± 10.40*
MPV (fl)	5.833 ± 3.17	7.933 ± 3.332	7.967 ± 3.811	9.367 ± 3.60
PDW%	15.70 ± 3.37	17.03 ± 3.547	18.83 ± 2.542	18.90 ± 3.318
PCT%	0.0733 ± 0.037	0.2667 ± 0.152	1.133 ± 0.513*	1.300 ± 0.692*
Neutrophils (%)	69.77 ± 2.55	78.27 ± 2.774	84.30 ± 3.996*	92.80 ± 4.850*
Lymphocytes (%)	26.83 ± 5.29	33.63 ± 3.465	41.67 ± 4.041*	52.93 ± 4.90*
Monocytes (%)	1.900 ± 0.65	3.967 ± 0.950	5.000 ± 1.00*	5.267 ± 1.350*
Eosinocytes (%)	2.367 ± 0.404	2.367 ± 0.3215	2.400 ± 0.30	2.400 ± 0.458

The data are represented as mean ± SD. Asterisk-bearing values show a significant difference (*p < *0.05) as compared to the control group.

### Histology

**Figure 5.**
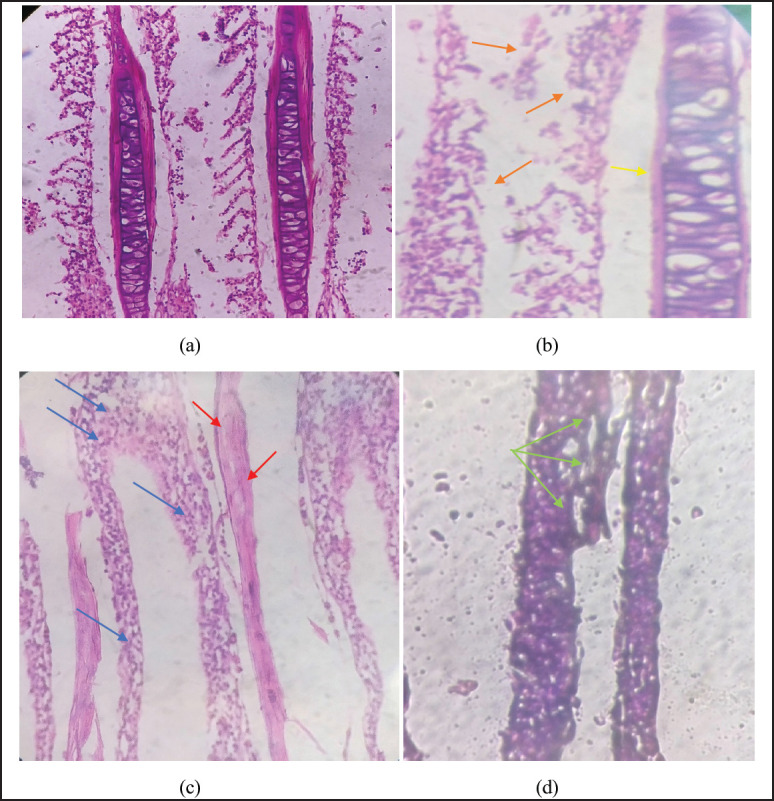
Showing histological alterations in the gills as (a) showing normal arrangement of gill filaments including primary and secondary lamellae, (b), (c), and (d) showing variations in gill tissues as tissue damage (orange arrow), nuclei cluster formation (blue arrow), and rupturing of gill lamellae (red arrow).

**Figure 6.**
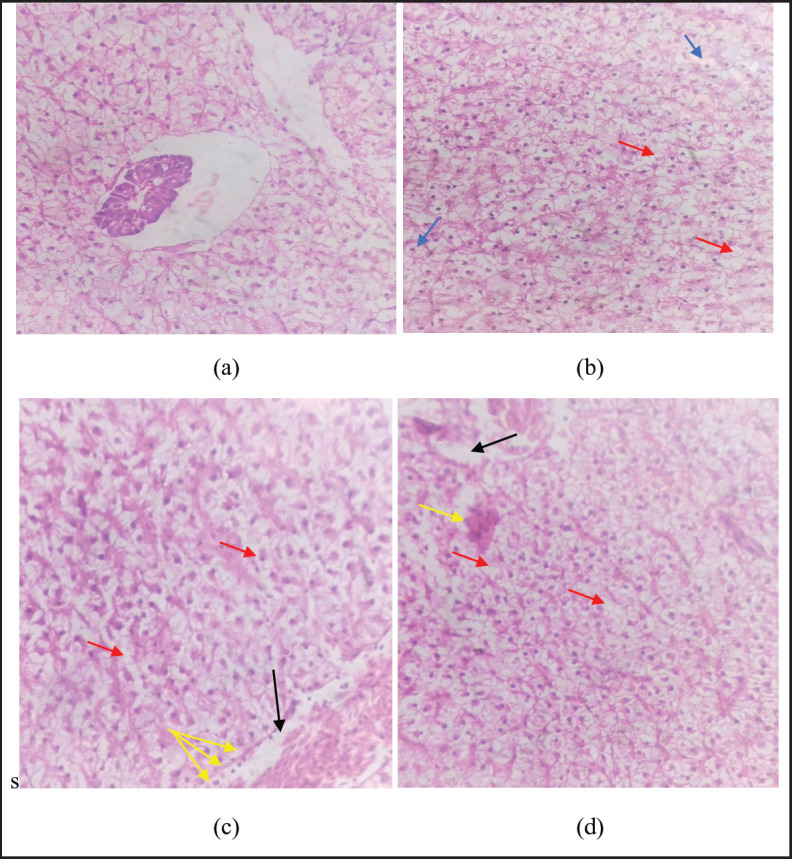
Showing histological alterations in the liver as (a) showing normal arrangement of hepatic cells in the control, (b), (c), and (d) showing necrosis in cells (red arrow), pyknotic nuclei (blue arrow), cluster nuclei formation (yellow arrow), and sinusoidal spaces (black arrow).

**Figure 7.**
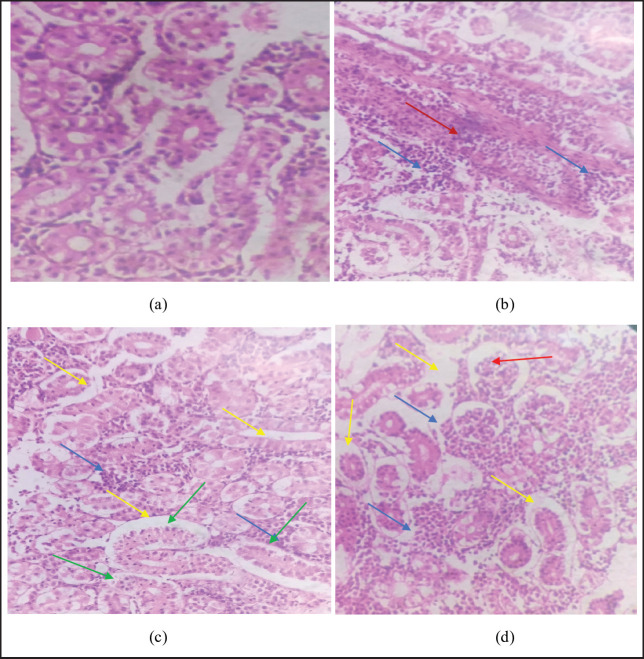
Showing histological alterations in the kidney tissues as (a) showing normal arrangement of renal cells, (b), (c), and (d) showing variations in parenchyma cells (dark red arrow), dilated renal tubules (green arrow), cluster nuclei formation (blue arrow), sinusoidal spaces (yellow arrow), and melanomacrophage (pale red arrow).

Figures 5, 6, and 7 display the histological alterations detected in the gills, kidneys, and liver of *L. rohita*. Figure 5a depicts the typical configuration of gill filaments, including primary and secondary lamellae, serving as a standard reference. Figure 5b–d displays the reference sample treated with smokeless nicotine. This treatment resulted in noticeable changes in the organization and spread of the primary and secondary gill lamellae. The observed alterations include tissue damage, swelling, the formation of clusters of nuclei, rupturing, and severe harm to both primary and secondary lamellae. Figure 6a illustrates the control group’s typical configuration of liver cells and the hepatic vein. Figure 6b–d displays necrosis in liver cells, pyknotic nuclei, clustered nuclei formation, and sinusoidal gaps. Figure 7a depicts the typical configuration of cells in the renal tissue of the control group. Figure 7b–d shows different histological alterations exposed to different concentrations of smokeless nicotine, including parenchymal cell formation, dilated renal tubules, nuclei cluster formation, sinusoidal spaces, and melanomacrophages.

## Discussion

Nicotine is a harmful and addictive drug [19]. Long-term exposure to nicotine causes hematological and biochemical alterations. Herxheimer et al. [20] reported that nicotine is responsible for the same alterations in hemodynamics that smoking cigarettes does. According to the present investigation, the concentrations of WBCs, HGB, RBCs, HCT, MCV, PCT, RDW-SD, neutrophils, lymphocytes, and monocytes were significantly increased as compared with the untreated group. Similar results are reported by Sharif et al. [21] and Dass et al. [22]. They also observed that nicotine causes elevations in WBC, HCT, and MCV concentrations. Aljarrah et al. [23] reported that nicotine causes the release of catecholamines, which leads to a rise in lymphocyte counts, which in turn causes an increase in white blood cell counts. Because nicotine has such a profound depressant impact on immunological function, the body responds by producing more WBCs to improve the immune system [24].

Our results showed an increased level of creatinine and a decreased level of blood urea. Similar findings were reported by Maeda et al. [25], which support our results. Creatinine is primarily a waste product of muscle breakdown, and it is eliminated from the muscle and into the blood. Blood creatinine and uric acid are useful indications of kidney health and renal function, as well as indicators for purine and muscle metabolism [26]. The high level of creatinine showed that, due to nicotine exposure, the breakdown of muscles increases [27]. Urea is the principal end product of catabolism. The low serum urea concentration may be attributable to the liver’s failure to metabolize protein [28]. Lipid profiles showed that cholesterol, triglycerides, LDL, and VLDL levels in the experimental groups were significantly increased as compared to the control group. These results were similar to those of Sharif et al. [21] in albino white rats. This increase is due to the activity of 3-hydroxy-3-methyl-glutaryl CoA reductase and the increased incorporation of labeled acetate in cholesterol. Chattopadhyay and Chattopadhyay [29] also observed high levels of cholesterol and triglycerides in the blood due to nicotine exposure. Nicotine reduces lipoprotein lipase activity, so the extrahepatic tissue does not take in as much circulating triglyceride-rich lipoprotein and very low-density lipoprotein (VLDL), leading to higher triglyceride levels [30].

Triglycerides are converted into glycerides and fatty acids by the process of lipolysis. Our results indicated that the administration of nicotine significantly increased ALT, AST, globulin, and blood glucose concentrations while causing a significant decrease *(p *≤ 0.05) in albumin and total protein levels. Similar results were reported by Jang et al. [31] and Sharif et al. [21]. Blood ALT and AST elevations are indicative of hepatic damage [32]. Glucose helps diagnose carbohydrate disorders. It is a reliable endocrine and physiological indication of the severity of several acute stressors [33]. The hyperglycemia in fish due to tobacco nicotine was also observed by Adamu [34]. This rise in serum glucose may be associated with stressful conditions caused by low levels of dissolved oxygen and a decrease in the activity of microorganisms involved in nicotine breakdown. According to the present study, nicotine exposure had no significant effect on T3, T4, or TSH concentrations. The effect of nicotine on metabolic hormones was also observed by Colzani et al. [35] and indicated the same results.

Histology was used as a very useful tool to provide information on the severity of tissue damage, injuries, and organ functionality [36]. In this study, histological changes were observed in the gills, kidneys, and liver of *L. rohita*. Histology of gills treated with smokeless nicotine showed alteration in the arrangement and distribution of primary and secondary gill lamellae, tissue damage, swelling, nuclei cluster formation, rupturing, and severe damage to primary and secondary lamellae. Awopetu and Modede [37] observed that nicotine caused damage to the gill filaments and the formation of lesions in the gills of catfish. Gills are essential for osmoregulation and breathing. Changes in gill structure reduce blood flow, causing problems with gas exchange and the maintenance of homeostasis in fish [28].

Histological analysis of the liver showed necrosis in hepatic cells, pyknotic nuclei, and sinusoidal spaces. No literature was found on the effects of smokeless nicotine on fish. Similar observations were reported by Kolure et al. [38] in rats. The liver is the major organ involved in nicotine metabolism. Iranloye and Bolarinwa [39] reported that nicotine causes histological alterations in the livers of rats. Liver enlargement may develop when lipids build up in the liver’s parenchyma cells due to illness, which indicates that nicotine may cause cellular damage that leads to lipid buildup in the hepatic cells. Kolure et al. [38], also reported that nicotine caused significant hepatotoxic effects in rats. The structure of a normal fish kidney consists of glomeruli inside the glomerular capsule, proximal tubules, and distal tubules wrapped in hematopoietic tissue. No histological changes were observed in the kidneys of the control fish group. Histology of the kidney showed different histological alterations exposed to different concentrations of smokeless nicotine, including parenchyma cell damage, dilated renal tubules, nuclei cluster formation, sinusoidal spaces, and melanomarophage. Similar results were also reported by Odokuma and Adogbeji [40] in rats. Exposure to nicotine causes deleterious effects on the kidney [41]. There is no previous literature found on the histological effects of smokeless nicotine powder on freshwater fish, *L. rohita,* and other fish.

## Conclusion

In conclusion, this study highlights the negative effects of nicotine on blood physiology and biochemical alterations in *L. rohita*. However, dose-dependent variations were observed. These changes included increased levels of HGB, RBCs, WBCs, HCT, MCV, RDW-SD, PCT, neutrophils, lymphocytes, and monocytes, as well as elevated levels of triglycerides, total cholesterol, LDL, VLDL, ALT, AST, globulin, thyroid stimulating hormone, BUN, creatinine, and blood glucose, while decreasing MCHC, MCH, RDW, and platelets, HDL, albumin, total proteins, and T3 levels. Histological alterations (tissue damage, swelling, nuclei cluster formation, pyknotic nuclei, and sinusoidal spaces) were also observed in the liver, gill, and kidney of exposed fish. These findings emphasize the harmful effects of nicotine on aquatic life and highlight the need for more strict measures to prevent its discharge into water bodies.

## List of Abbreviations

ALT, alanine transaminase; AST, aspartate aminotransferase; HGB, hemoglobin; HDL, high-density lipoprotein; HCT, hematocrit; MCH, mean corpuscular hemoglobin; MCV, mean corpuscular volume; MCHC, mean corpuscular hemoglobin concentration; PCT, procalcitonin; RBCs, red blood cells; RDW, red cell distribution width; LDL, low-density lipoprotein; T4, tetraiodothyronine; TSH, thyroid-stimulating hormone; VLDL, very low-density lipoprotein; WBCs, white blood cells
